# Circulating neutrophil-to-lymphocyte ratio at admission predicts the long-term outcome in acute traumatic cervical spinal cord injury patients

**DOI:** 10.1186/s12891-020-03556-z

**Published:** 2020-08-15

**Authors:** Jian-Lan Zhao, Song-Tao Lai, Zhuo-Ying Du, Jian Xu, Yi-Rui Sun, Qiang Yuan, Xing Wu, Zhi-Qi Li, Jin Hu, Rong Xie

**Affiliations:** 1 Department of Neurosurgery, Huashan Hospital, Shanghai Medical College, Fudan University, 12 Wulumuqi Road (M), Shanghai, 200040 P.R. China; 2 Neurosurgical Institute of Fudan University, 12 Wulumuqi Road (M), Shanghai, 200040 P.R. China; 3 Shanghai Clinical Medical Center of Neurosurgery, 12 Wulumuqi Road (M), Shanghai, 200040 P.R. China; 4 Shanghai Key laboratory of Brain Function and Restoration and Neural Regeneration, 12 Wulumuqi Road (M), Shanghai, 200040 P.R. China; 5 Department of Radiation Oncology, Fudan University Shanghai Cancer Center, Department of Oncology, Shanghai Medical College, Fudan University, Shanghai, 200032 China; 6 Department of General Surgery, the Seventh Affiliated Hospital, SUN Yat-sen University, Shenzhen, 518000 China

**Keywords:** Acute traumatic cervical spinal cord injury, Neutrophil-to-lymphocyte ratio, Prediction model, Prognostic value, 6-months outcome

## Abstract

**Background:**

The prognostic value of Neutrophil-to-Lymphocyte Ratio (NLR) for the outcome of acute cervical traumatic spinal cord injury (tSCI) patients has rarely been studied by now throughout the world.

**Methods:**

We performed a single-center retrospective cohort study to evaluate the prognostic value of NLR from peripheral whole blood count in patients with acute cervical tSCI. Patients within 6 h of acute cervical tSCI treated between Dec 2008 and May 2018 in Huashan Hospital of Fudan University were enrolled. Outcomes of patients with tSCI were assessed using American spinal injury association Impairment Scale (AIS). 6-month outcomes were dichotomized into poor outcome group (AIS A to C) and good outcome group (AIS D and E). Uni- and multivariate analyses were performed to assess the independent predictors of 6-month outcome. Two prediction models based on admission characteristics were built to evaluate the prognostic value of NLR. The discriminative ability of predictive models was evaluated using the area under the curve (AUC).

**Results:**

A total of 377 patients were identified from our single center in China PR. Multivariate analysis showed that age, AIS grade at admission, NLR (*p* < 0.001) and coagulopathy (*p* = 0.003) were independent predictors of the 6-months outcome for acute cervical tSCI patients. The model combing NLR and standard variables (AUC = 0.944; 95% CI, 0.923–0.964) showed a more favorable prognostic value than that without NLR (AUC = 0.841; 95% CI, 0.798–0.885) in terms of 6-month outcome.

**Conclusions:**

NLR is firstly identified as an independent predictor of the 6-month outcome in acute cervical tSCI patients worldwide. The prognostic value of NLR is favorable, and a high NLR is associated with poor outcome in patients with acute cervical tSCI.

## Background

Traumatic spinal cord injury (tSCI), especially the cervical spinal cord, is one of the most devastating form of trauma because of its high morbidity rate and enormous financial and social burden. The prevalence of tSCI is approximately 750 per million worldwide with a trend of rising annual incidence [1]. Similar to traumatic brain injury (TBI), the primary injury of spinal cord is induced by trauma impact, while the secondary injury is triggered by multiple factors during different time courses after injury, among which the inflammatory response plays a vital role [2, 3].

It was recognized that either primary or secondary injury was associated with the outcome of tSCI patients was considered to be [4, 5], and several standard prognostic factors at admission, including age, blood cells counts, coagulation status, Charleson Co-morbidity Index (CCI), American spinal injury association Impairment Scale (AIS) grades, and the initial Glasgow Coma Scale (GCS) score, were identified to be associated with the outcome of tSCI patients [6, 7]; however, their prognostic values were limited even a variety of predictive models were built. Thus, to develop a model with higher prognostic value is justified.

As mentioned previously, inflammation responses, which are considered to contribute to the secondary tSCI, are partly activated by purinergic signaling in the acute phase [8]. After focal tSCI, circulating neutrophils can be observed to be recruited to the site of injury within 1 h, which is considered as an indicator of acute inflammation [9]. It was reported that high neutrophils and low lymphocytes level in the peripheral blood at admission were independently associated with poor outcome of intracerebral hemorrhage (ICH) patients, and the neutrophil-to-lymphocyte ratio (NLR) was readily available as an outcome predictor [10, 11]. Nevertheless, the prognostic value of NLR for the outcome of tSCI patients has rarely been studied. Moreover, whether combining NLR with standard independent predictors would improve prognostic is still unknown.

Our study evaluated the prognostic value of NLR and tested its predictive power in prediction models in terms of the 6-months outcome for cervical tSCI patients.

## Methods

### Study design and participants

We performed a retrospective, observational cohort study in Huashan Hospital of Fudan University. This study was approved by the Institutional Review Board of Huashan Hospital of Fudan University. Between Dec. 2008 and May. 2018, patients with acute tSCI who were admitted to the Department of the Neurosurgery were recruited. Written informed consent was obtained from all individual participants.

Inclusion criteria were set as follow: 1) patients with a diagnosis of traumatic spinal cord injury confirmed by computed tomography (CT); 2) more than 14 years of age; 3) within 6 h after injury; 4) initial GCS > 13; 5) initial AIS grade A to D and 6) C1 to T1segment spinal injury. Exclusion criteria included: 1) patients with traumatic injury to a body region other than the cervical spinal cord with an Abbreviated Injury Severity (AIS) score ≥ 3; 2) a penetrating neck injury; 3) pregnancy; 4) pre-injury major neurologic deficits or disease (i.e. ischemic stroke, Parkinson’s Disease) and 5) injury related medical treatments, including methylprednisolone, spinal surgeries and et al., in other hospitals or medical centers before admission. All patients were evaluated and treated by full-time neurosurgeons with specific training in critical care.

### Blood cells count and demographic data

At admission, blood samples of included patients were collected and analyzed by the Central Clinical Chemistry Laboratory of Huashan Hospital. White blood cells (WBC), neutrophil ratio and lymphocyte ratio were routinely performed in all patients within 6 h of injury. Neurologic examinations were performed according to the standards established by the American Spinal Injury Association (ASIA).

Clinical and demographic characteristics, including age, gender, types of injury, Charleson Co-morbidity Index (CCI), initial Glasgow Coma Scale (GCS) score, and the overall ASIA Impairment Scale (AIS) grade were recorded for all patients. Injury types were assessed by using initial CT scans on admission.

Coagulopathy was defined as our previous studies [12, 13]. Briefly, platelet counts (PLT) < 100 × 10^9^/L, international normalized ratio (INR) > 1.25, prothrombin time (PT) > 14 s, and activated partial thromboplastin time (APTT) > 36 s.

### Outcome assessments

The primary outcome was ordinal change in AIS grade at 6 months after injury, which was in accordance to the recommendations by the National Acute Spinal Cord Injury Study (NASCIS). Moreover, several notable natural history studies and clinical trials such as Sygen trial has already demonstrated that the vast majority of patients showed signs of neurological recovery during this period. We carefully assessed the 6-months outcome of SCI patients via outpatient interviews or over the phone after discharge. The outcome of included patients was dichotomized into poor (AIS A to C) and good outcome (AIS D and E) [14, 15]. Relevant postoperative complications were also recorded.

### Predictive models and statistical analysis

The prognostic models derived from the data of included patients. Two models for 6-months outcome were developed based on admission characteristics: model A included standard predictors such as age, gender, mechanism of injury, AIS grades at admission, and types of injury; model B included the results of WBC counts, neutrophil ratio, lymphocyte ratio and NLR in addition to the predictors in model A.

Continuous variables/predictors were expressed as means ± standard deviation (SD) or medians (interquartile range) and categorical variables as percentages. The univariate analyses of categorical data were performed using the chi-squared test. Equality of variance was assessed using Levene’s test. Normally distributed variables were compared using Student’s t-tests or one-way ANOVA, whereas non-normally distributed variables were compared using the Kruskal-Wallis or Mann–Whitney U-tests.

Following the univariate analyses, a forward stepwise logistic regression analysis of the 6-months outcome was used to develop the prediction models and adjust for multiple predictors of 6-months outcome. By constructing receiver operative curve (ROC) and calculating the area under the curve (AUC), we evaluated the specificity and sensitivity of the two models, and their discriminative power. All statistical tests were two-tailed and *p* values < 0.05 were considered statistically significant. Statistical analysis was performed using SPSS 23.0 (IBM, USA) and MedCalc statistical software (version 15.2.2, MedCalc Software bvba, Ostend, Belgium).

## Results

**Fig. 1:**
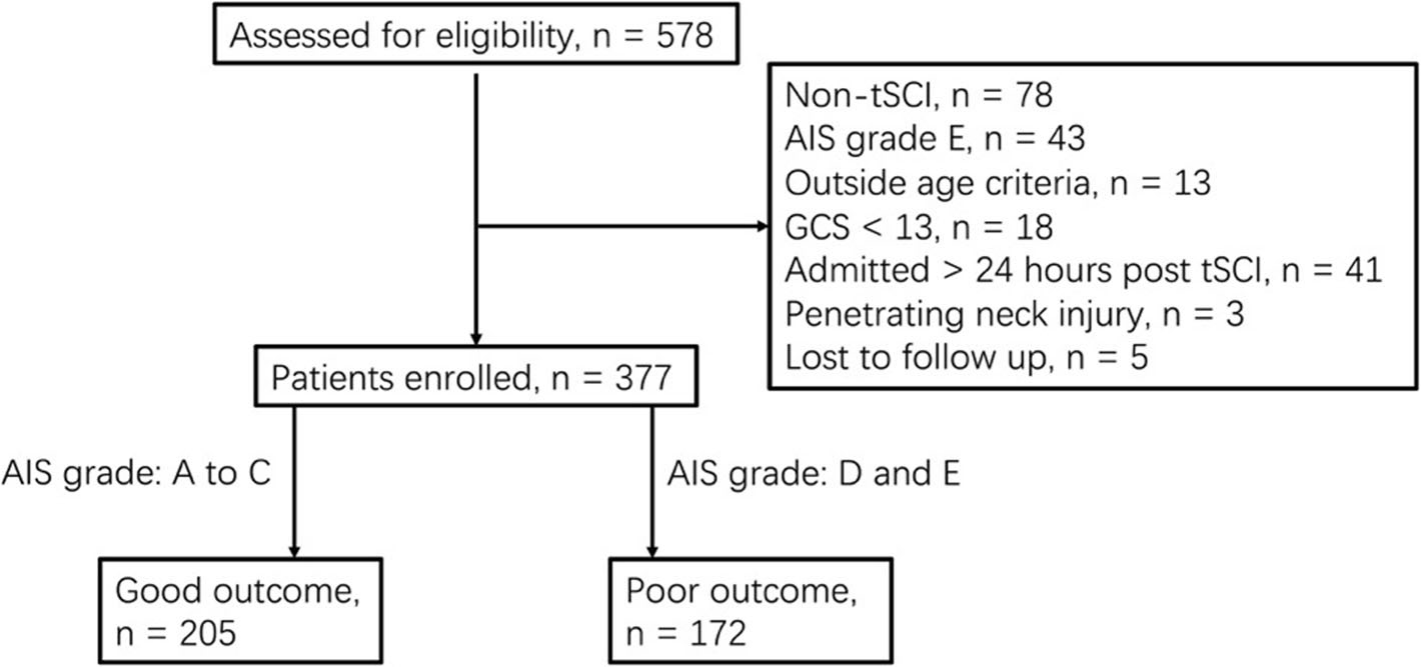
Flowchart of patients’ selection

A total of 377 cervical tSCI patients were included in our study. The flowchart of participants’ selection is shown as Fig. 1. Favorable outcome was confirmed in 205 (54.4%) patients among the 377 included patients.

The univariate analysis revealed that age, baseline AIS grade, and coagulopathy were significantly related with the 6-months outcome (*p* < 0.001). Meanwhile, WBC counts, neutrophil ratio, lymphocyte ratio and NLR also showed close correlation with the outcome of cervical tSCI patients (p < 0.001). Patients with poor outcome had a significantly higher WBC counts, neutrophil ratio, NLR and lower lymphocyte ratio than those with good outcome (Table 1).

**Table 1:** Baseline Characteristics According to the 6-months Outcome

	Full cohort	Poor outcome (AIS A to C)	Good outcome (AIS D to E)	P value
N	377	172	205	
Age (yrs) (mean ± SD)	46.05 ± 17.93	52.91 ± 13.61	44.61 ± 16.34	< 0.001
Male (N, %)	212 (56.2)	102 (59.3)	110 (48.9)	0.298
Mechanism of injury (n, %)				0.762
Motor vehicle accident	87 (23.1)	38 (22.1)	49 (23.9)	
Fall	99 (26.3)	51 (29.7)	48 (23.4)	
Stumble	102 (27.1)	49 (28.5)	53 (25.8)	
Blow to spine	68 (18.0)	31 (18.1)	37 (18.1)	
Others	21 (5.5)	8 (4.6)	13 (6.3)	
GCS at admission	14.8 ± 0.4	14.7 ± 0.4	14.8 ± 0.3	0.562
Baseline AIS grade (n, %)				
A	129 (34.2)	80 (46.5)	49 (23.9)	< 0.001
B	61 (16.2)	48 (27.9)	13 (6.3)	< 0.001
C	72 (19.1)	51 (29.6)	21 (10.2)	< 0.001
D	115 (30.5)	3 (1.7)	112 (54.6)	< 0.001
Coagulopathy (n, %)	87 (23.1)	59 (34.3)	28 (13.7)	< 0.001
Charleson Co-morbidity Index > 1 (n, %)	79 (20.9)	41 (20.9)	38 (20.7)	0.253
WBCs, (×10^9^/L)	14.68 ± 5.19	19.01 ± 6.32	13.18 ± 4.59	< 0.001
Neutrophil ratio	0.87 ± 0.09	0.89 ± 0.09	0.81 ± 0.05	< 0.001
Lymphocyte ratio	0.13 ± 0.06	0.09 ± 0.03	0.12 ± 0.07	< 0.001
NLR	13.28 ± 11.46	25.73 ± 11.36	7.98 ± 7.31	< 0.001

Data are given as mean ± SD, or n (%) unless otherwise noted

The degree of neurologic improvement was measured by changes in AIS grade from admission to 6 months follow-up (Table 2). AIS grade improvements of the 377 included patients were as follows: 159 (42.2%) had no improvement, 148 (39.2%) had a 1 grade improvement, 55 (14.6%) had a 2 grades improvement, 9 (2.4%) had a 3 grades improvement and 6 (1.6%) had a 1 grade worsening.

**Table 2:** Ordinal changes in AIS grade from admission to 6 months follow-up

AIS grade at admission	A	B	C	D	E	Total
A	67	34	22	6	0	129
B	2	13	19	24	3	61
C	0	1	14	48	9	72
D	0	0	3	65	47	115

*AIS* American spinal injury association Impairment Scale (AIS)

To analyze and adjust for multiple predictors, we further performed a forward stepwise logistic regression analysis. After adjustment, NLR remained a statistically significant prognostic factor of 6-months outcome of cervical tSCI patients (OR, 0.93; 95% CI, 0.87–0.98; *p* < 0.001), while WBC counts, neutrophil ratio and lymphocyte showed no significant correlation with patients’ 6-months outcome (Table 3). Other independent prognostic factors included age, baseline AIS grade (p < 0.001) and coagulopathy (*p* = 0.003) (Table 4).

**Table 3:** Associations of Leukocyte Counts and Neutrophil-to-Lymphocyte Ratio (NLR) with the 6-months outcome of tSCI patients

Independent Variable	Unadjusted		Adjusted	
	OR (95% CI)	P Value	OR (95% CI)	P Value
WBCs (×1,000/mm^3^)	0.82 (0.77–0.83)	< 0.001	1.02 (0.94–1.07)	0.156
Neutrophil ratio	0.76 (0.72–0.81)	< 0.001	0.91 (0.82–1.01)	0.168
Lymphocyte ratio	0.82 (0.74–0.93)	< 0.001	1.11 (1.04–1.22)	0.103
NLR	0.83 (0.77–0.90)	< 0.001	0.93 (0.87–0.98)	< 0.001

**Table 4:** Multivariate logistic regression analysis predicting the 6-months outcome

Independent variable	Adjusted OR (95% CI)	p value
Age	0.91 (0.86–0.97)	< 0.001
GCS at admission	1.18 (0.93–1.34)	0.713
Charleson Co-morbidity Index > 1	1.02 (0.88–1.16)	0.328
Coagulopathy	0.84 (0.64–0.97)	0.003
Baseline AIS grade		
A	1	–
B	0.51 (0.39–0.81)	< 0.001
C	0.59 (0.28–0.72)	< 0.001
D	0.62 (0.41–0.93)	< 0.001
Neutrophil-to-lymphocyte ratio	0.93 (0.87–0.98)	< 0.001

*CI* confidence interval. *OR* odds ratio. The reference category value was transformed from 0 to 1 to compare subgroups

**Fig. 2:**
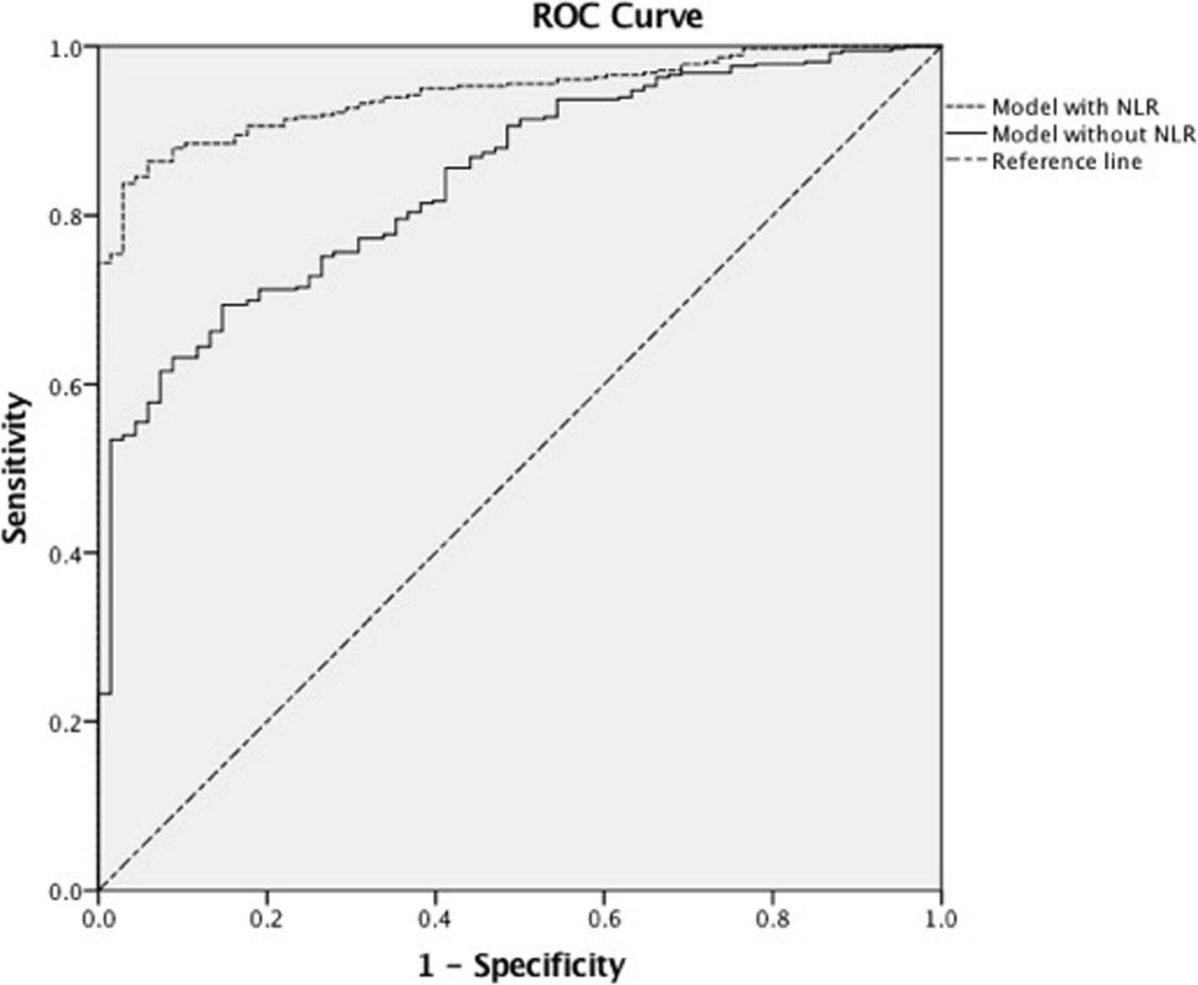
Receiver operative curve (ROC) of the two predictive models. The model with Neutrophil-to-Lymphocyte Ratio (NLR) had a larger are under the curve (AUC). It is indicated that the discrimination of the model with NLR is more favorable than the other two models

Lastly, we developed two predictive models for the 6-months outcome. To assess their discriminative ability, we constructed ROC and calculated AUC (Fig. 2). It is indicated that the predictive model with NLR (AUC = 0.944; 95% CI, 0.923–0.964) showed a more favorable discrimination than that of the model without NLR (AUC = 0.841; 95% CI, 0.798–0.885). Thus, the prognostic ability of NLR to the 6-months outcome of cervical tSCI patients is favorable.

## Discussion

The main finding of our study was that cervical tSCI patients with lower 6-months AIS grades (A to C) showed significantly higher circulating NLR than those with higher AIS grades (D or E), and the level of circulating NLR was an independent prognostic factor of 6-months outcome of cervical tSCI patients. Moreover, by calculating AUC, we found that the predictive model, combining age, gender, mechanism of injury, coagulation status and NLR, showed good discrimination than models based on solely on standard predictors. Thus, it is inferred that the predictive power of standard model can be improved by circulating NLR in patients with acute cervical spinal cord injury.

For that outcome prediction at admission is important for cervical tSCI patients, NLR has rarely been studied as a prognostic factor for cervical tSCI patients. Predictive models can promote quality control through the standardization of the parameters for patient assessment which can be compared across physicians and institutions [16]. The prognostic value of predictors is determined by their reliability on assessment, the prevalence of abnormalities, and the strength of the prognostic effect [17]. Predictors we used in this study, including age, gender, coagulation status and components of circulating blood sample, can be readily obtained on admission, and their prognostic value had previously been confirmed either in tSCI or TBI patients [6, 17–19]. The counts of WBCs and its components, including neutrophils ratio, lymphocytes ratio and NLR, are readily available lab tests with standardized results, although rarely studied, it is reasonable to include these results into this prognostic model.

Favorable prognostic value of circulating NLR at admission had been reported in ICH and TBI patients [11, 20]. It is expected that the prognostic value of NLR would be favorable in predicting the outcome of patients with cervical tSCI which shares the similar secondary injury mechanism as TBI. Both Uni- and multi-variate analysis showed significant correlation between NLR and patient outcome, and the prognostic effect remained substantial following adjusted analysis, which suggesting NLR is of considerable prognostic relevance in cervical tSCI patients.

As the importance of circulating blood components in predicting outcome is increasingly recognized, a variety of parameters, as monocyte to HDL cholesterol ratio, [21] and LDL-C/HDL-C ratio [22] have been studied and applied in predicting the outcome of patients with acute ischemic or hemorrhagic cerebral diseases. NLR, which conveys crucial information about the complex inflammatory activity in vascular bed, is an established marker of systemic inflammation and is easily calculated [23]. In ICH patients, a high NLR at admission was associated with poor outcome, but its underlying mechanism remained unclear [11]. In the current study, cervical tSCI patients who had a higher NLR were more likely to have a poor outcome measured by AIS grade, which is consistent with results from ICH or TBI patients [12]. In addition, the level of NLR is similar with that in TBI patients, but much higher than the level in ICH patients. It is assumed that unlike the primary injury in ICH, which is mainly induced by focal hematoma compression, primary injury in tSCI is more massive and diffuse, including the spinal cord laceration and contusion, resulting more severe acute inflammatory responses. Being considered as an indicator of severity level of acute inflammatory responses, the neutrophil counts will increase more dramatically compared with other leukocytes after spinal cord injury. Increasing of lymphocyte indicated the occurrence of chronic inflammations or a virus infection, in the other word, after acute tSCI, lymphocyte counts may will not dramatically increase. As a result, NLR level increases at early stage after spinal cord injury and is much higher than that in ICH patients.

It was recognized that secondary inflammatory injury was significantly associated with poor outcome in traumatic central nervous system (CNS) injury patients [3, 24, 25]. And its potential mechanism may be evolved with damage-associated molecular patterns (DAMP) released after CNS injury, and inflammatory response may initially be triggered by these DAMP [26, 27]. Moreover, significant leukocytes infiltration can be observed after CNS injury, and thus, focal inflammatory response can be aggravated, and finally, worsened inflammatory related CNS injury [28]. In the case of acute tSCI, the neutrophils are actively recruited around injury site, and contribute to the cellular injury and disruption of viable spinal cord tissues. Dramatic increase of neutrophils is seen as early as within the first hour of injury, while that of lymphocyte shows no significant changes.

Our study had several limitations. Firstly, the time course of our study was relatively long, thus the level of emergency may be different. Secondly, we included only cervical tSCI, thus a further study is required to clarify such assumption in patients with lumbar or thoracic injury. Thirdly, the predictive ability of inflammation markers, such as C-reactive protein et al., might also play a role in outcome prediction, which would be investigated in our future studies. Last but not least, a prospective multi-center study is justified to further elucidate the relationship of NLR and 6-months outcome of patients with tSCI, moreover, a survival statistic is also required,

## Conclusion

NLR is firstly identified as an independent predictor of the 6-month outcome in acute cervical tSCI patients worldwide. The prognostic value of NLR is favorable, and a high NLR is associated with poor outcome in patients with acute cervical tSCI.

## Abbreviations

AIS: American spinal injury association Impairment Scale
APTT: Activated partial thromboplastin time
AUC: Area under the curve
CCI: Charleson Co-morbidity Index
CT: Computed tomography
GCS: Glasgow Coma Scale
INR: International normalized ratio
NLR: Neutrophil-to-Lymphocyte Ratio
OR: Odds ratios
PLT: Platelet counts
tSCI: traumatic spinal cord injury
WBC: White blood cell
